# Relation between Maximum Oral Muscle Pressure and Dentoalveolar Characteristics in Patients with Cleft Lip and/or Palate: A Prospective Comparative Study

**DOI:** 10.3390/jcm12144598

**Published:** 2023-07-10

**Authors:** Lisa Van Geneugden, Anna Verdonck, Guy Willems, Greet Hens, Maria Cadenas de Llano-Pérula

**Affiliations:** 1 Department of Oral Health Sciences-Orthodontics, KU Leuven, University Hospitals Leuven, Kapucijnenvoer 7, Blok 1, Bus 7001, 3000 Leuven, Belgium; lisa.vangeneugden@uzleuven.be (L.V.G.); an.verdonck@uzleuven.be (A.V.); guy.willems@uzleuven.be (G.W.); 2 Department of Neurosciences, KU Leuven, University Hospitals Leuven, Kapucijnenvoer 7, Blok H, Bus 7001, 3000 Leuven, Belgium; greet.hens@uzleuven.be

**Keywords:** cleft lip and palate, cleft palate, orthodontics, palatal expansion, crossbite, muscle activity

## Abstract

Background: Perioral muscle function, which influences maxillofacial growth and tooth position, can be affected in patients with oral clefts due to their inherent anatomical characteristics and the multiple surgical corrections performed. This research aims to (1) compare the maximum oral muscle pressure of subjects with and without isolated cleft palate (CP) or unilateral cleft lip and palate (UCLP), (2) investigate its influence on their dentoalveolar characteristics, and (3) investigate the influence of functional habits on the maximum oral muscle pressure in patients with and without cleft. Material and methods: Subjects with and without CP and UCLP seeking treatment at the Department of Orthodontics of University Hospitals Leuven between January 2021 and August 2022 were invited to participate. The Iowa Oral Performance Instrument (IOPI) was used to measure their maximum tongue, lip, and cheek pressure. An imbalance score was calculated to express the relationship between tongue and lip pressure. Upper and lower intercanine (ICD) and intermolar distance (IMD) were measured on 3D digital dental casts, and the presence of functional habits was reported by the patients. The data were analyzed with multivariable linear models, correcting for age and gender. Results: 44 subjects with CP or UCLP (mean age: 12.00 years) and 104 non-affected patients (mean age: 11.13 years) were included. No significant differences in maximum oral muscle pressure or imbalance score were detected between controls and clefts or between cleft types. Significantly smaller upper ICDs and larger upper and lower IMDs were found in patients with clefts. A significant difference between controls and clefts was found in the relationship between oral muscle pressure and transversal jaw width. In cleft patients, the higher the maximum tongue pressure, the wider the upper and lower IMD, the higher the lip pressure, the smaller the upper and lower ICD and IMD, and the higher the imbalance score, the larger the upper and lower IMD and lower ICD. An imbalance favoring the tongue was found in cleft patients. The influence of functional habits on the maximum oral muscle pressure was not statistically different between clefts and controls. Conclusion: Patients with CP or UCLP did not present reduced maximum oral muscle pressure compared with patients without a cleft. In cleft patients, tongue pressure was consistently greater than lip pressure, and those who presented a larger maxillary width presented systematically higher imbalance scores (favoring the tongue) than those with narrow maxillae. Therefore, the influence of slow maxillary expansion on maximum oral muscle pressure in cleft patients should not be underestimated.

## 1. Introduction

An appropriate lip and tongue function is indispensable for speech and non-verbal oral functions such as swallowing. Large discrepancies in the activity of perioral muscles such as the orbicularis oris, buccinator, or tongue can affect oral aesthetics and ultimately contribute to a deterioration of the patient’s oral health-related quality of life (OHRQoL; Table 1 includes a description of abbreviations and acronyms used) [1].

The balance between intra- and extra-oral muscles is believed to influence maxillofacial growth and the position of the teeth [2]. Lip and tongue function affects the form of the dental arch [3] and has been linked with occlusal traits such as overjet and incisor inclination [4,5,6], the growth of the cranial base [4], and vertical skeletal facial patterns [5,6]. In addition, functional asymmetries of masticatory muscles can affect postural equilibrium by disturbing the neck and facial muscle balance [7]. Therefore, alterations in muscle strength and activity may result in dental and/or skeletal disharmony and discrepancies in facial morphology.

In patients with cleft lip and/or palate, perioral muscle function could be restricted due to the cleft affecting the anatomy of some of these muscles. Additionally, the multiple surgical corrections performed on these patients in order to attempt reconstruction of the oral muscles could influence both their aesthetics and function [7].

Nevertheless, there is limited research regarding oral muscle function in patients with cleft lip and/or palate. The scarce evidence reports a reduced range of upper lip movement [8] and the presence of compensatory movements of the lower lip and chin in patients with clefts [9], as well as increased upper lip function and compression of the lips during swallowing [10]. Normal lip and tongue function in 25 patients with UCLP aged 6.7 to 18.2 years has also been reported [11]. However, the literature focuses almost exclusively on unilateral cleft lip and palate [9,12,13,14], includes very small control groups of unaffected patients [13,14], and research regarding tongue and cheek pressure is scarce [9,12]. Additionally, the balance between tongue and lip function has been barely investigated [5]. In patients with anatomical muscle conditions such as cleft lip and/or palate, it could be of added value to not only look at tongue and lip function separately but also at how they both work in relation to each other.

Therefore, this research aims to (1) compare the maximum tongue, lip, and cheek muscle pressure and the imbalance of intra and extraoral muscles of subjects with and without CP or UCLP; (2) investigate the influence of the maximum oral muscle pressure and muscle imbalance on the dentoalveolar characteristics; and (3) examine the influence of functional habits on the maximum oral muscle pressure of both groups.

## 2. Materials and Methods

The protocol of this prospective comparative study was defined prior to the start and approved by the Ethics Committee of University Hospitals Leuven and KU Leuven University, Belgium, with registration number s64829. Informed consent was obtained from all the participating subjects and their parents/guardians before enrollment in the study.

### 2.1. Subject Recruitment

In the cleft group, subjects with CP and UCLP, under the age of 18 and of self-reported European descent, seeking treatment at the Department of Oral Health Sciences—Orthodontics of University Hospitals Leuven between January 2021 and August 2022 were invited to participate. Subjects presenting only submucosal clefts, only clefts of the lip or the alveolar bone, bilateral cleft lip and palate, the Robin sequence, and have a confirmed diagnosis of a syndromic disorder or pathogenic mutations were excluded.

All the included cleft patients were operated on by the same two surgeons at the same hospital with the following surgical protocol: In patients with UCLP, lip closure was performed at the age of 3 months using a modified Millard’s technique. In patients with CP and UCLP, soft palate closure took place at 12 months-old by using a modified Veau–Wardill–Kilner push-back technique with supraperiosteal dissection and hard palate closure between the ages of 5 and 7 years in order to minimize maxillary growth restriction due to scar tissue by using the modified von Langenbeck procedure. It is important to remark that cleft patients undergo orthodontic maxillary expansion on two occasions: before the hard palate closure and before the secondary alveolar bone graft, normally planned around the age of 9 to facilitate the eruption of the maxillary canines.

In the control group, subjects without oral clefts, muscular diseases, or syndromes, aged under 18 years and of self-reported European descent, seeking treatment at the same department between October 2021 and November 2021, were invited to participate. This control group, composed at random over two months, represents the ‘average’ orthodontic patient.

### 2.2. Assessment of Dentoalveolar Data

Dental alginate impressions, a wax bite registration, and intra- and extraoral photographs were taken from all patients as part of the standard orthodontic diagnosis since all included subjects needed orthodontic treatment. The dental impressions and the wax bite registration were then digitalized, and the 3D digital dental casts were analyzed using the software DigiModel^®^ (OrthoProof^®^ version 3.46.2, Nieuwegein, The Netherlands). The following parameters were measured on the casts: intercanine and intermolar distance (ICD and IMD, as described by Van de Velde et al. [15]), overjet, overbite, and the presence of a crossbite. The sagittal occlusion of canines and first molars was noted by using an ordinal system where 0 was defined as neutro-occlusion, –1.00 as a full premolar width mesio-occlusion, and +1.00 as a full premolar width disto-occlusion. Intervals of 0.25 were used to express all intermediate occlusions. The measurements were taken before the start of comprehensive orthodontic treatment (including functional or fixed appliances) for all patients.

### 2.3. Assessment of Functional Habits

The following functional habits were clinically evaluated: the patient’s swallowing pattern (mature or infantile), the position of the tongue at rest (physiological, interdental, or caudal), and the competence of the lips (competent or not). Additionally, the following parafunctions were verbally asked about and, if possible, clinically checked: nail biting, pen chewing, thumb sucking, and bruxism.

### 2.4. Assessment of Oral Muscle Pressure

Perioral muscle pressure was measured with the Iowa oral performance instrument (IOPI) [16,17], which is a widely used, validated tool. It is easy to use in children, and it keeps the diagnostic procedure as short as possible. It measures the amount of pressure a certain muscle exerts by means of an air-filled bulb. Anterior tongue pressure was measured by placing the bulb on the midline of the tongue and instructing the patient to push it against the palate at the height of the anterior rugae as hard as possible. Lip pressure was measured by positioning the air-filled bulb between two wooden blades. These were then placed between the lips at the region of the incisors, and the patient was asked to close the lips as hard as possible. Right and left cheek pressure were measured by placing the bulb between the cheek and the canine-premolar region and then asking the patient to squeeze as hard as possible. All measurements lasted up to 10 s and were repeated three times with a resting period of 30 s between each trial. The maximum values of the tongue, lip, right, and left cheek pressure were recorded. The measurements were taken by five senior orthodontic residents, trained and calibrated in advance. The measurements were taken before the start of comprehensive orthodontic treatment (including functional or fixed appliances) for all patients. The intra-observer reliability of the myofunctional outcome parameters (maximum tongue, lip, and cheek pressure) was calculated by using the intra-class correlation coefficient (ICC) for each of the 5 observers.

An imbalance score, previously described in the literature [5], was used to represent the imbalance between lip and tongue pressure. The score was calculated as follows:

$$\frac{(\mathrm{maximum}\;\mathrm{tongue}\;\mathrm{pressure}-\mathrm{maximum}\;\mathrm{lip}\;\mathrm{pressure})\times 100}{\mathrm{maximum}\;\mathrm{tongue}\;\mathrm{pressure}+\mathrm{maximum}\;\mathrm{lip}\;\mathrm{pressure}}.$$

### 2.5. Statistical Analysis

The first aim of this research was to compare the maximum tongue, lip, and cheek pressure of subjects with and without cleft lip and/or palate. For this, multivariable linear models were used, modeling the muscle pressure as the response variable and the type of patient (cleft or non-cleft) as the explanatory variable of interest. Age and gender were included as covariates to account for possible confounding.

For the comparison of the transversal width (ICD and IMD) in the upper and lower jaws of subjects with and without clefts, a series of *t*-tests were used for pairwise comparison.

To investigate the influence of oral muscle pressure on dentoalveolar characteristics in patients with and without cleft (second aim), multivariable linear models were also used, and again, age and gender were included as covariates to account for possible confounding. The dentoalveolar characteristics were divided into two big groups: transversal width (ICM and IMD) and occlusal parameters (occlusion on the left and right sides, overjet, overbite, and crossbite), with these dentoalveolar characteristics as the response variable. The type of patient (cleft or non-cleft), muscle pressure, and the interaction between both terms were included in the explanatory model. A similar analysis was performed, defining the three patient groups as control, CP, or UCLP.

The third aim was to investigate the influence of functional habits on oral muscle pressure in patients with and without cleft palates. Multivariable linear models were also used, and age and gender were included as covariates to account for possible confounding. The regression analyses modeled muscle pressure as the response variable, type of patient (cleft or non-cleft), functional habits (dichotomous variable), and the interaction between both terms in the explanatory model.

Analyses were performed with SAS software (version 9.4 of the SAS System for Windows). A probability level of <0.05 was considered significant.

## 3. Results

Forty-four cleft patients were included (13 girls and 31 boys, mean age 12.00 years old, range 7.06–16.57 years old). 16 and 28 subjects presented CP and UCLP, respectively. Of the CP patients, 7 presented a cleft of the soft palate only. From the UCLP subjects, 17 presented the cleft on the right side, and 11 presented Simonart’s band. Three of the UCLP patients had undergone presurgical nasoalveolar molding (PNAM), and one UCLP patient had used lip straps before the surgical lip closure.

The control group consisted of 104 patients (53 girls and 51 boys, mean age 11.13 years old, range 7.17–13.97 years old). Table 2 presents the demographic information of the included sample.

**Figure 1:**
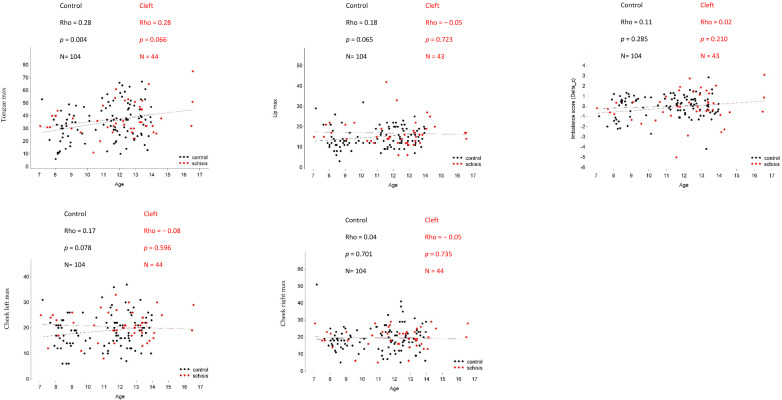
Scatter plots of the strength of the association between oral muscle pressure and age, quantified by Pearson correlation coefficients.

Gender distribution was significantly different between the cleft and non-cleft groups (*p* = 0.019, Fisher’s Exact test), which was expected since isolated CP is more common in women while UCLP occurs more frequently in men [18]. The mean age of the cleft group was nearly 1 year older than the non-affected group (*p* = 0.026, Mann–Whitney U test). Since literature shows that oral strength differs between males and females and that it increases with age in healthy subjects [19], age and gender were included as covariates in the statistical model to account for possible confounding. Figure 1 shows the strength of the association between myofunctional outcomes and age, quantified by Pearson correlation coefficients and visualized by means of scatter plots. The only association that could be demonstrated was an increase with age in maximum tongue pressure in the control group.

**Figure 2:**
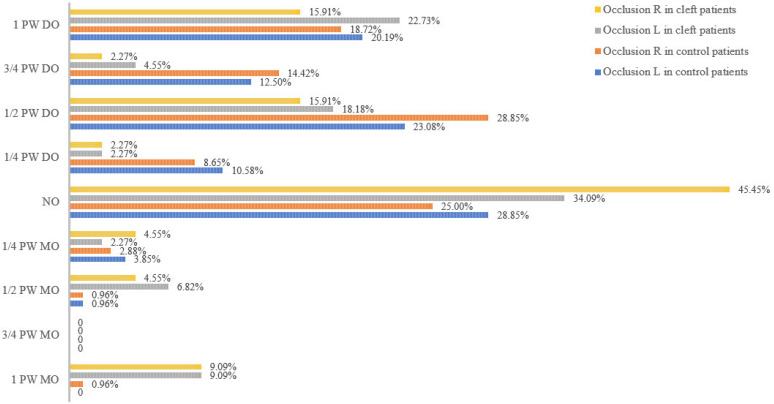
Distribution of the occlusion (on the left and right side) in both the cleft and control group.

The distribution of the occlusion in both the cleft and control groups is shown in Figure 2. The fact that control patients also present malocclusion was a conscious choice since comparing patients with cleft lip and/or palate to an ‘ideal’ group without malocclusion will be more likely to show differences. Most patients presented a Class II malocclusion (66.35–70.64% controls, 37.73–36.36% clefts, left and right, respectively), followed by a Class I malocclusion (28.85–25.00% controls, 34.09–45.45% clefts, left and right, respectively). Finally, 4.81% (left side) and 4.80% (right side) of the control patients and 18.18% (left side) and 18.19% (right side) of the cleft patients presented a Class III malocclusion.

Of all 148 included participants, one subject failed to have the lip function properly measured, which was considered a missing value. The mean intra-observer reliability (ICC) of the myofunctional outcome parameters (maximum tongue, lip, and cheek pressure) of the 5 observers was 0.803.

Table 2 also shows the mean values of muscle pressure and imbalance score in each group. Table 3 shows the statistical significance and the mean differences in muscle pressure and imbalance scores between the groups. Positive mean differences correspond with higher values for the first group compared to the second one, and vice versa. No significant differences in maximum oral muscle pressure or imbalance score were detected between controls and clefts, nor between cleft types.

Table 4 reports the values of maxillary width per group (intercanine and intermolar distances). Results show significantly smaller upper ICDs and larger upper and lower IMDs in patients with clefts compared to controls, and larger lower ICDs and upper IMDs in patients with CP compared to controls. The upper ICD was smaller and the lower IMD was larger in patients with UCLP compared to the control group. No significant differences in ICD or IMD were found between patients with CP and UCLP.

Table 5 reports whether there is a relation between jaw width (ICD and IMD) and perioral muscle pressure (tongue, lip, and cheek pressure and imbalance score) within and between the different groups. Positive slope values refer to positive relations (higher muscle pressure values for larger width values). A significant relationship was found between maximum anterior tongue pressure and jaw width in cleft patients. The higher the maximum tongue pressure, the wider the upper and lower IMD in patients with clefts compared to controls. The same relationship was found for CP patients compared to controls. For UCLP patients, the wider the upper and lower ICDs. Additionally, the higher the tongue pressure, the wider the upper ICD in patients with UCLP compared to patients with CP. Lip pressure was also significantly related to jaw width. The higher the lip pressure, the smaller the upper and lower ICD and IMD in patients with cleft and CP (compared to controls). In patients with UCLP, the effect of lip pressure on the upper ICD and lower IMD was not statistically significant. Regarding the imbalance score, the higher it was in cleft patients, the larger the upper and lower IMD and lower ICD. In patients with CP, the higher the imbalance score, the larger the upper and lower IMD. Furthermore, in UCLP patients, the higher the imbalance score, the larger the upper and lower IMD and ICD. An imbalance favoring the tongue was observed in patients with CP and UCLP. A significant relationship was found between cheek pressure and transversal width. The higher the right cheek pressure in patients with CP and UCLP and the higher the left cheek pressure in patients with UCLP, the smaller the upper ICD. The higher the right cheek pressure, the smaller the lower IMD in patients with CP compared to patients with UCLP.

Table 6 shows the relation between muscle pressure and the remaining occlusal characteristics (sagittal molar occlusion, overjet, overbite, and crossbite) within and between the groups. Results for ordinal outcomes are presented as slopes indicating the change in dental outcome for a 1 unit increase in perioral muscle pressure. Positive slope values refer to positive relations (higher muscle pressure values for larger occlusal values). Results for cross-bite as a binary outcome are presented as odds ratios (OR), indicating the change in odds for cross-bite for a 1 unit increase in perioral muscle pressure. OR > 1 refers to positive relations (higher muscle pressure values for higher odds of a crossbite). Age and gender were included as covariates to account for possible confounding.

Statistically significant differences were found between the control and cleft groups regarding maximum tongue pressure on the sagittal (right and left) occlusion, whereby a larger tongue pressure led to a tendency towards mesio-occlusion in patients with cleft compared to controls. The effect of maximum lip pressure was significantly different between controls versus cleft, controls versus CP, and controls versus UCLP for the right sagittal occlusion, whereby a larger lip pressure led to a tendency towards disto-occlusion in the cleft group. On the left side, the same effect was seen in patients with UCLP compared to controls. This can be explained by the increased occurrence of the unilateral variant on the right side. Significant differences were also observed between clefts and controls regarding the effect of the imbalance score on right occlusion: the higher the imbalance score in cleft patients (more tongue than lip pressure), the greater the tendency to mesio-occlusion. Additionally, the higher the imbalance score, the greater the tendency towards mesio-occlusion on the right and left sides in patients with UCLP compared to controls. A significant difference was seen between controls and CP and between CP and UCLP regarding overbite: the higher the imbalance score, the more tendency there is towards an open bite in CP. Significant differences were found between controls and UCLP regarding the effect of maximum right and left cheek pressure on overjet: the higher the cheek pressure, the more negative the overjet (and thus, a greater tendency towards reverse overjet). A significant difference was seen between controls and cleft patients regarding right cheek pressure: the higher the right cheek pressure in cleft patients, the higher the chance of a cross bite.

Finally, the influence of the observed functional habits on the maximum oral muscle pressure was statistically different between cleft and controls (Table 7), but this significance presented a very wide range of 95% CI and was not considered valuable. Within the control group, infantile swallowing and the interdental position of the tongue at rest significantly affected maximum tongue pressure. The caudal and interdental positions of the tongue at rest also significantly affected the imbalance score.

## 4. Discussion

This prospective study measures the maximum pressure of the lips, tongue, and cheeks in 44 subjects with CP and UCLP, comparing it with that of a non-affected control group consisting of 104 subjects, which is a very large control group in comparison to similar studies [9,12,13,14]. The data was collected before the start of comprehensive orthodontic treatment. Results show that there is no significant difference in maximum tongue, lip, or cheek pressure or imbalance score between subjects with and without clefts. Our findings are similar to those from the study of Van Lierde et al. [11], which compares the oral muscle pressure of 25 patients with UCLP with that of 25 control patients, also using the IOPI and reporting normal lip and tongue pressure. However, a comparison between patients with CP and UCLP has never been made in the literature before. Our results show no significant differences in maximum oral muscle pressure between patients with CP and UCLP. This suggests that the involvement of the lip and alveolar bone in patients with UCLP does not necessarily affect maximum oral muscle pressure and that cleft repair surgery performed at a young age ensures a good functional recovery of the muscles.

Important to note is that 11 of the 28 included UCLP patients (39%) had a Simonart’s band on the cleft side, which according to the literature has a minor positive effect on the facial pattern, reduces the need for the secondary nose and lip repair [20], and requires less traumatic lip and palate repair [21]. Furthermore, three of the UCLP patients had undergone PNAM, which improves nasal asymmetry [22], prevents the positioning of the tongue between the palatal shelves [23], molds the protruded premaxilla, and reduces the size of the cleft lip, alveolus, and palate [22]. One UCLP patient had used presurgical lip straps to facilitate lip closure [23]. These presurgical techniques could influence our outcome since they aim to reduce the width of the defect and the lip tension.

It is relevant to take into consideration that all cleft patients included in this study were operated on by the same two surgeons at the same hospital, following the same surgical protocol. However, between 2012 and 2013, a new surgical protocol for lip and palate repair was gradually implemented by the Cleft Team of University Hospitals Leuven. In the new surgical protocol, the hard palate is closed using a vomer flap at the time of lip repair. The repair of the soft palate still takes place at 10–12 months old, but no longer by supra-periosteal push-back; radical intravelar veloplasty as described by Sommerlad [24] is used instead. In this new protocol, cleft patients undergo an orthodontic expansion only before the secondary alveolar bone graft. Research is ongoing to compare both surgical protocols and examine if the new surgical techniques cause less scar tissue and have a positive influence on oral muscle pressure in cleft patients.

The present study also explores the influence of the maximum oral muscle pressure on jaw width, measured by ICD and IMD. Significantly smaller upper ICDs and larger upper and lower IMDs were found in cleft patients compared with controls. It is important to remark that 19 of the 44 included cleft patients (43,18%) (5 with CP and 14 with UCLP) had previously undergone interceptive orthodontic treatment with removable expansion plates, while only 8 of the 104 control patients (7.69%) had undergone interceptive expansion of the maxilla, which can influence the outcome. Early orthodontic treatment in cleft patients often overexpands the upper jaw, having more effect at the height of the molars than the canines [25], since the canines are positioned closer to the cleft and thus also to the scar tissue. However, it is practically impossible to recruit a sample of cleft patients that did not undergo early maxillary expansion since the lack of growth in the maxillary suture and the presence of scar tissue on the palate lead to maxillary growth impairment [26]. Early orthodontic expansion is then performed in preparation for the closure of the hard palate and the secondary alveolar bone graft. The fact that the lower intermolar distance was larger in patients with clefts in comparison to patients without clefts may be due to a lower position of the tongue in patients with clefts [27].

Furthermore, since we measured jaw width with 32 dental landmarks depending on the dentition stage, we can also make statements regarding tooth position. For example, it can be concluded that the higher the tongue pressure, the more the permanent molars are inclined buccally in patients with CP. This is also true for the canines and molars in the upper jaw in UCLP patients.

The functional harmony and balance of the perioral muscles are critical not only in craniofacial growth and development but also in the position and inclination of the teeth. According to the equilibrium theory, teeth receive intraoral and extraoral muscle forces [28]. A lack of balance in these muscle pressures would affect the occlusion and, in turn, craniofacial growth. However, occlusal forces may also need to be taken into account, as well as the presence of functional habits [4,28,29,30,31]. In this study, a significant difference in tongue and lip pressure on the transversal width was found between clefts and controls. This significant relationship means that the intra-oral muscles increase the transversal width, and the extra-oral muscles would cause a decrease in the transversal width since the pressure comes from the outside towards the teeth. In patients with a cleft, the higher the imbalance score, the higher the transversal width, meaning that intra-oral forces are more prominent than extra-oral forces. In other words, in cleft patients, there is an imbalance favoring the tongue. Literature has described that the forces from the tongue are consistently greater than those from the lips in non-affected patients [4,28,29,30,31]. The results of this study suggest that this phenomenon could be extended to the cleft population.

However, perioral muscle pressure at rest is considered to be more crucial than that exerted during function [4,32], since forces at rest are exerted for much longer periods of time [17]. Several authors have attempted to measure oral muscle pressure at rest with various devices, but this was found to be very challenging [33,34]. Measurements during functional activities such as swallowing are also demanding because the procedure of swallowing itself is very complex, consists of different steps [33], and depends on parameters such as the type of device used [35], the type of bolus [36], the amount of occlusal units [35], etc. Maximum tongue pressure is a simpler measurement to take, is less dependent on other external factors, can be compared between studies and/or patients, is correlated with the swallowing function [35], and has been used as a diagnostic feature to detect muscular diseases [37,38].

This study also investigates the potential relationship between oral muscle pressure and sagittal occlusion, overjet, overbite, and cross bite in patients with and without clefts. The effect of the tongue on sagittal occlusion was significantly different between patients with and without cleft: in cleft patients, a greater tongue pressure led to more mesio-occlusion. Patients with cleft palate often present a skeletal and dental class III relationship due to the underdevelopment of the maxilla [39], caused by the lack of transversal growth in the midpalatal suture and the presence of scar tissue on the palate [25]. Pereira et al. [27] stated that patients with class III malocclusion exhibit recessed tongue positioning on the floor of the mouth. Our results confirm these findings and suggest that this effect will be amplified with higher maximum tongue pressure. However, according to Wen-hua et al. [2], children with class III have fewer perioral forces, while Lambrechts et al. [40] found no significant relationship between class III and lip and tongue forces in non-affected patients. It would be very interesting for future research to compare the oral muscle pressure of class III patients with and without cleft palate, which has not yet been performed in the literature.

The effect of the maximum lip pressure on the occlusion was significantly different between the controls and clefts: a greater lip pressure led to more disto-occlusion on the right side in the total cleft group, the CP group, and the UCLP group compared to the control group. The same effect was seen on the left side in the UCLP group compared to the controls. This can be explained by the increased occurrence of the unilateral variant on the right side. The findings of the cleft group could be due to compensatory movements of the lower lip, a more prominent lower lip, or a higher stomion position. The literature reports both a reduced range of upper lip movement [13] and compensatory movement of the lower lip [9] in cleft patients. Since the IOPI does not discriminate between upper and lower lip pressure, these aspects could not be explored in our study.

The present research found a significant difference between the cleft and control groups regarding the right cheek pressure: the higher the right cheek pressure, the greater the chance of a cross bite being present. This could be explained by the presence of a cleft on the right side in 17 of the 28 UCLP patients. Literature is controversial on this topic. While Kecik et al. [41] also found higher masseter activity at the cross bite side in patients without cleft, Szyszka-Sommerfeld et al. [42] found lower masticatory muscle activity in cleft patients. However, the literature does agree with the fact that orthodontic correction of a cross bite is essential to restore the balance of the oral muscles [43].

The present study also looked into the relationship between maximum oral muscle pressure and functional habits in control and cleft patients. The influence of the swallowing pattern, resting position of lips and tongue, and several parafunctions on the maximum oral muscle pressure was not statistically different between the groups.

In addition, it would also have been useful to know whether the patients had undergone speech therapy since this could influence the myofunctional results and can be seen as a limitation. Furthermore, the presence of scar tissue at the level of the lips and the palate, as well as the shape of the palate, could also play a role in the measurement of tral muscle pressure. These aspects were also not investigated in this study.

Finally, the results of this study may have some clinical implications regarding early orthodontic expansion in patients with CP and UCLP. Maxillary expansion in patients with clefts is essential as it contributes to speech development, masticatory function, tooth eruption, and stabilization of the dental arches. However, maxillary expansion has been reported to relapse in 80% of patients with cleft [44,45], since they are especially susceptible to occlusal instability due to congenitally missing teeth, a lack of bone in the cleft site [45,46], and the presence of surgical scar tissue. For these reasons, some studies recommend compensatory overexpansion [47] or prolonging the retention period for 2-3 years [48]. However, our results suggest that cleft patients with a larger maxillary width present a higher imbalance score favoring the tongue than those with a narrower maxilla. Since almost half of the included cleft patients had previous interceptive orthodontic treatment with removable expansion plates, we can conclude that maxillary orthodontic expansion has an important influence on the maximum oral muscle pressure. For this reason, attention should be paid to the expansion protocols, for instance, by optimizing the design of the appliance, the amount of expansion, and the retention phase. It could also be an option to revise the retention protocols for patients with cleft palates. Hawley retainers could be additionally equipped with a transversal screw or extra springs, which could be activated in case of relapse of the upper transversal width. Retainers could be supplemented by myofunctional trainers in order to improve the oral muscle function of cleft patients and prevent relapse after orthodontic expansion, keeping the palate as wide as possible. Speech therapy could also be integrated before, during, and after orthodontic treatment [49]. However, the motivation and burden of care for the patient have to be taken into account.

## 5. Conclusions

Patients with cleft lip and/or palate did not present reduced maximum oral muscle pressure compared with patients without this condition. An imbalance favoring the tongue (the tongue pressure is greater than the lip pressure) was found in both patients with and without clefts. Patients with CP and UCLP with a larger transversal maxillary width presented systematically higher imbalance scores (favoring the tongue) than those with narrow maxillae. Therefore, the influence of slow maxillary expansion on maximum oral muscle pressure in cleft patients should not be underestimated. It is important for cleft teams to take these myofunctional aspects into consideration in the treatment of patients with cleft lip and/or palate, especially regarding orthodontic treatment planning.

## Figures and Tables

**Table 1:** List of abbreviations and acronyms used in this article.

Abbreviations	Definition
OHRQoL	Oral Health-related Quality of Life
CP	Isolated cleft palate
UCLP	Unilateral cleft lip and palate
ICD	Intercanine distance
IMD	Intermolar distance
IOPI	Iowa Oral Performance Instrument
LCD	Liquid crystal display
CI	Confidence interval
SAS	Statistical Analysis System
PNAM	Presurgical nasoalveolar moulding
kPa	Kilopascal
13g–23g	Distance between the most lingual and cervical points of the permanent maxillary canines, if present
13c–23c	Distance between the cusps of the permanent maxillary canines, if present
16g–26g	Distance between the most lingual and cervical points of the permanent maxillary first molar, if present
16f–26f	Distance between the central fossae connected to the vestibular groove of the permanent maxillary first molar if present
16MB–26MB	Distance between mesio-buccal cusps of the permanent maxillary first molar, if present
16DB–26DB	Distance between disto-buccal cusps of the permanent maxillary first molar, if present
33g–43g	Distance between the most lingual and cervical points of the permanent mandibular canines, if present
33c–43c	Distance between the cusps of the permanent mandibular canines, if present
36g–46g	Distance between the most lingual and cervical points of the permanent mandibular first molar, if present
36f–46f	Distance between the central fossae connected to the vestibular groove of the permanent mandibular first molar, if present
36MB–46MB	Distance between the mesio-buccal cusps of the permanent mandibular first molar, if present
36DB–46DB	Distance between the disto-buccal cusps of the permanent mandibular first molar, if present

**Table 2:** Demographic information of the sample, maximum tongue, lip, right, and left cheek pressure in kilopascals (kPa), and imbalance scores of the included subjects.

		Control	CP + UCLP	CP	UCLP
General information	N	104	44	16	28 (17 right)
	Mean age (years)	11.13	12.00	12.50	11.72
	Age range (years) SD (years)	7.17–13.97 1.948	7.06–16.57 2.409	8.04–14.60 1.702	7.06–16.57 2.721
	Girls (n/N (%))	53 (50.96%)	13 (29.55%)	9 (56.25%)	4 (14.29%)
	Boys (n/N (%))	51 (49.04%)	31 (70.45%)	7 (43.75%)	24 (85.71%)
Tongue pressure	N	104	44	16	28
	Mean	35.00	37.95	34.88	39.71
	SD	14.19	12.50	11.71	13.07
Lip pressure	N	104	43	16	27
	Mean	15.09	16.74	17.56	16.26
	SD	5.86	6.41	8.44	4.97
Left cheek pressure	N	104	44	16	28
	Mean	18.92	20.39	21.63	19.68
	SD	6.61	6.61	5.83	6.17
Right cheek pressure	N	104	44	16	27
	Mean	19.30	19.48	20.81	18.71
	SD	7.44	6.41	5.47	6.87
Imbalance score	N	104	43	16	27
	Mean	36.98	37.31	32.37	40.25
	SD	20.634	21.493	26.577	17.74

**Table 3:** Mean differences in muscle pressure between the groups, as well as the statistical significance. Positive mean differences correspond with higher values for the first group compared to the second one, and vice versa. Age and gender were included as covariates to account for possible confounding.

Muscle Pressure	CP + UCLP vs. Control		CP vs. Control		UCLP vs. Control		CP vs. UCLP	
	Mean Diff (95% CI)	*p* Value	Mean Diff (95% CI)	*p* Value	Mean Diff (95% CI)	*p* Value	Mean Diff (95% CI)	*p* Value
Tongue pressure	0.957 (−3.919; 5.833)	0.698	−2.627 (−9.783; 4.530)	0.469	3.137 (−2.680; 8.955)	0.288	−5.764 (−14.21; 2.680)	0.179
Lip pressure	1.965 (−0.243; 4.173)	0.081	1.913 (−1.297; 5.123)	0.240	1.999 (−0.676; 4.673)	0.142	−0.086 (−3.905; 3.733)	0.965
Left cheek pressure	1.018 (−1.357; 3.394)	0.398	2.441 (−1.052; 5.934)	0.169	0.153 (−2.687; 2.993)	0.915	2.288 (−1.834; 6.410)	0.274
Right cheek pressure	0.144 (−2.510; 2.798)	0.915	1.525 (−2.383; 5.432)	0.442	−0.695 (−3.872; 2.481)	0.666	2.220 (−2.391; 6.831)	0.343
Imbalance score	−2.975 (−10.62; 4.668)	0.443	−6.331 (−17.42; 4.754)	0.261	−0.813 (−10.05; 8.422)	0.862	−5.519 (−18.71; 7.669)	0.410

**Table 4:** Intercanine and intermolar distance of the included subjects, as well as the statistical significance (pairwise comparison between the four groups) according to a series of *t*-tests. A probability level of <0.05 was considered significant (* *p* < 0.05).

Maxilla										Mandible									
		Control (1)	CP + UCLP (2)	CP (3)	UCLP (4)	P 1–2	P 1–3	P 1–4	P 3–4			Control (1)	CP + UCLP (2)	CP (3)	UCLP (4)	P 1–2	P 1–3	P 1–4	P 3–4
13g–23g	N	62	21	12	9						N	64	27	13	14				
	Mean	26.66	25.05	25.77	24.08	0.171	0.452	0.083	0.349	33g–43g	Mean	20.57	21.25	21.91	20.63	0.170	0.047 *	0.923	0.225
	Std	3.903	3.993	2.497	5.426						Std	2.204	2.694	2.104	3.096				
13c–23c	N	62	21	12	9						N	64	27	13	14				
	Mean	32.74	30.41	32.29	27.92	<0.001 *	0.584	<0.001 *	0.078	33c–43c	Mean	26.06	25.64	26.06	25.25	0.552	1.000	0.286	0.539
	Std	2.497	5.634	2.828	7.491						Std	2.119	3.305	2.483	3.977				
16g–26g	N	103	36	14	22						N	102	35	13	22				
	Mean	32.21	33.81	34.37	33.45	0.030 *	0.011 *	0.093	0.543	36g–46g	Mean	32.62	34.10	33.80	34.28	0.020 *	0.111	0.007 *	0.687
	Std	2.749	4.321	4.061	4.535						Std	2.373	3.315	3.321	3.377				
16f-26f	N	103	36	14	22						N	102	35	13	22				
	Mean	43.78	45.13	45.78	44.72	0.140	0.041 *	0.283	0.563	36f-46f	Mean	40.67	41.63	40.81	42.11	0.113	0.865	0.027 *	0.366
	Std	3.210	5.253	4.680	5.655						Std	2.416	4.051	4.335	3.896				
16MB–26MB	N	103	36	14	22						N	102	35	13	22				
	Mean	48.38	48.03	48.64	47.64	0.660	0.806	0.366	0.579	36MB–46MB	Mean	43.93	44.39	43.45	44.95	0.310	0.579	0.155	0.330
	Std	3.165	5.140	5.825	4.757						Std	2.694	4.331	4.486	4.243				
16DB–26DB	N	102	35	13	22						N	101	35	13	22				
	Mean	50.50	50.81	51.36	50.48	0.731	0.397	0.989	0.627	36DB–46DB	Mean	46.21	47.39	46.68	47.80	0.152	0.630	0.047 *	0.456
	Std	3.190	5.066	5.229	5.063						Std	3.192	4.205	4.370	4.152				

Abbreviations: 13g–23g: distance between the most lingual and cervical points of the permanent maxillary canines if present; 13c–23c: distance between the cusps of the permanent maxillary canines if present; 16g–26g: distance between the most lingual and cervical points of the permanent maxillary first molar if present; 16f-26f: distance between the central fossae connected to the vestibular groove of the permanent maxillary first molar, if present; 16MB–26MB: distance between mesio-buccal cusps of the permanent maxillary first molar, if present; 16DB–26DB: distance between disto-buccal cusps of the permanent maxillary first molar, if present; 33g–43g: distance between the most lingual and cervical points of the permanent mandibular canines if present; 33c–43c: distance between the cusps of the permanent mandibular canines, if present; 36g–46g: distance between the most lingual and cervical points of the permanent mandibular first molar, if present; 36f-46f: distance between the central fossae connected to the vestibular groove of the permanent mandibular first molar, if present; 36MB–46MB: distance between the mesio-buccal cusps of the permanent mandibular first molar, if present; 36DB–46DB: distance between the disto-buccal cusps of the permanent mandibular first molar, if present.

**Table 5:** Relation between jaw width (ICD and IMD) and perioral muscle pressure (tongue, lip, and cheek pressure and imbalance score). Within-group results are presented as slopes indicating the change in dental outcome for a 1 unit increase in perioral muscle pressure. Positive slope values refer to positive relations (higher muscle pressure values for larger width values). Age and gender were included as covariates to account for possible confounding. *p*-values for differences between slopes between groups are presented in the last 4 columns. A probability level of <0.05 was considered significant (* *p* < 0.05).

	Jaw Width	Control		CP + UCLP		CP		UCLP		Control vs. CP + UCLP	Control vs. CP	Control vs. UCLP	CP vs. UCLP
		Slope (95% CI)	*p*-Value	Slope (95% CI)	*p*-Value	Slope (95% CI)	*p*-Value	Slope (95% CI)	*p*-Value	Test for Difference of Slopes between Groups *p*-Value			
Effect of tongue pressure	13g–23g	−0.041 (−0.111; 0.030)	0.252	0.028 (−0.114; 0.171)	0.697	−0.044 (−0.255; 0.167)	0.680	0.219 (−0.015; 0.454)	0.066	0.391	0.978	0.037 *	0.101
	13c–23c	−0.012 (−0.111; 0.030)	0.703	0.023 (−0.106; 0.153)	0.720	−0.034 (−0.209; 0.141)	0.700	0.332 (0.138; 0.526)	0.001 *	0.625	0.815	0.001 *	0.007 *
	16g–26g	0.027 (−0.017; 0.070)	0.229	0.092 (0.009; 0.174)	0.029 *	0.173 (0.022; 0.324)	0.025 *	0.076 (−0.026; 0.177)	0.142	0.170	0.068	0.382	0.292
	16f-26f	−0.004 (−0.056; 0.048)	0.883	0.132 (0.034; 0.229)	0.008 *	0.194 (0.014; 0.374)	0.035 *	0.130 (0.010; 0.251)	0.035 *	0.016 *	0.038 *	0.045 *	0.562
	16MB–26MB	0.033 (−0.017; 0.084)	0.197	0.126 (0.031; 0.221)	0.010 *	0.309 (0.136; 0.481)	<0.001 *	0.068 (−0.048; 0.184)	0.245	0.092	0.003 *	0.583	0.024 *
	16DB–26DB	0.006 (−0.045; 0.057)	0.820	0.129 (0.034; 0.225)	0.008 *	0.296 (0.122; 0.470)	0.001 *	0.079 (−0.037; 0.196)	0.180	0.025*	0.002 *	0.254	0.043 *
	33g–43g	−0.007 (−0.047; 0.033)	0.730	0.046 (−0.017; 0.110)	0.150	0.042 (−0.070; 0.154)	0.455	0.092 (0.007; 0.177)	0.034 *	0.162	0.412	0.038 *	0.482
	33c–43c	−0.013 (−0.056; 0.030)	0.552	0.054 (−0.014; 0.121)	0.121	0.000 (−0.119; 0.120)	0.995	0.117 (0.027; 0.208)	0.012 *	0.104	0.836	0.011 *	0.124
	36g–46g	0.007 (−0.029; 0.042)	0.705	0.105 (0.038; 0.171)	0.002 *	0.076 (−0.048; 0.200)	0.226	0.120 (0.037; 0.203)	0.005 *	0.012 *	0.289	0.015 *	0.561
	36f–46f	0.010 (−0.027; 0.047)	0.603	0.173 (0.103; 0.243)	<0.001 *	0.189 (0.059; 0.320)	0.005 *	0.163 (0.076; 0.251)	<0.001 *	<0.001 *	0.010 *	0.002 *	0.742
	36MB–46MB	0.011 (−0.030; 0.053)	0.600	0.167 (0.089; 0.245)	<0.001 *	0.214 (0.069; 0.358)	0.004 *	0.140 (0.043; 0.237)	0.005 *	<0.001 *	0.009 *	0.017 *	0.406
	36DB–46DB	−0.003 (−0.049; 0.042)	0.885	0.182 (0.097; 0.268)	<0.001 *	0.211 (0.052; 0.369)	0.010 *	0.170 (0.064; 0.276)	0.002 *	<0.001 *	0.012 *	0.004 *	0.674
	13g–23g	−0.057 (−0.230; 0.115)	0.508	−0.232 (−0.591; 0.127)	0.202	−0.308 (−0.767; 0.150)	0.184	−0.104 (−0.691; 0.483)	0.725	0.385	0.311	0.880	0.586
	13c–23c	0.013 (−0.139; 0.165)	0.866	−0.378 (−0.695; −0.061)	0.020 *	−0.444 (−0.830; −0.059)	0.024 *	−0.263 (−0.756; 0.231)	0.293	0.030 *	0.030 *	0.290	0.565
	16g–26g	0.087 (−0.020; 0.194)	0.110	−0.190 (−0.394; 0.014)	0.068	0.000 (−0.338; 0.339)	0.999	−0.285 (−0.543; −0.027)	0.031 *	0.019*	0.630	0.009 *	0.187
	16f–26f	0.034 (−0.095; 0.163)	0.602	−0.202 (−0.448; 0.044)	0.107	0.042 (−0.365; 0.450)	0.838	−0.326 (−0.637; −0.016)	0.040 *	0.095	0.969	0.036 *	0.157
	16MB–26MB	0.109 (−0.012; 0.230)	0.078	−0.393 (−0.625; −0.162)	0.001 *	−0.320 (−0.706; 0.067)	0.104	−0.423 (−0.717; −0.128	0.005 *	<0.001 *	0.038 *	0.001 *	0.675
	16DB–26DB	0.072 (−0.052; 0.196)	0.251	−0.320 (−0.560; −0.080)	0.009 *	−0.266 (−0.691; 0.159)	0.219	−0.335 (−0.635; −0.035)	0.029 *	0.005 *	0.134	0.015 *	0.794
	33g–43g	0.020 (−0.080; 0.120)	0.695	−0.220 (−0.377; −0.064)	0.006 *	−0.129 (−0.394; 0.135)	0.334	−0.252 (−0.449; −0.055)	0.013 *	0.012 *	0.298	0.016 *	0.462
	33c–43c	−0.032 (−0.136; 0.071)	0.539	−0.306 (−0.468; −0.145)	<0.001 *	−0.282 (−0.559; −0.005)	0.046 *	−0.313 (−0.519; −0.107)	0.003 *	0.006 *	0.050 *	0.018 *	0.859
	36g–46g	0.140 (0.017; 0.191))	0.012 *	−0.071 (−0.240; 0.097)	0.403	−0.075 (−0.358; 0.207)	0.600	−0.076 (−0.289; 0.138)	0.484	0.070	0.234	0.126	0.997
	36f–46f	0.095 (−0.005; 0.194)	0.063	−0.175 (−0.360; 0.011)	0.065	−0.355 (−0.662; −0.048)	0.024 *	−0.091 (−0.323; 0.141)	0.438	0.013 *	0.007 *	0.147	0.177
	36MB–46MB	0.137 (0.029; 0.245)	0.014 *	−0.162 (−0.363; 0.040)	0.115	−0.302 (−0.636; 0.032)	0.076	−0.130 (−0.356; 0.149)	0.419	0.011 *	0.015 *	0.086	0.350
	36DB–46DB	0.100 (−0.019; 0.219)	0.099	−0.215 (−0.437; 0.007)	0.057	−0.353 (−0.723; 0.017)	0.061	−0.154 (−0.433; 0.125)	0.277	0.015 *	0.023 *	0.100	0.398
	13g–23g	−0.024 (−0.087; 0.040)	0.455	0.029 (−0.055; 0.112)	0.497	0.013 (−0.091; 0.116)	0.809	0.114 (−0.044; 0.271)	0.155	0.322	0.551	0.111	0.290
	13c–23c	−0.028 (−0.085; 0.028)	0.279	0.061 (−0.013; 0.135)	0.104	0.056 (−0.029; 0.140)	0.193	0.194 (0.065; 0.322)	0.004 *	0.059	0.095	0.002 *	0.078
	16g–26g	0.003 (−0.027; 0.033)	0.837	0.078 (0.025; 0.131)	0.004 *	0.076 (−0.003; 0.155)	0.059	0.087 (0.014; 0.159)	0.020 *	0.016 *	0.090	0.037 *	0.844
	16f–26f	−0.000 (−0.036; 0.035)	0.987	0.097 (0.034; 0.160)	0.003 *	0.084 (−0.010; 0.178)	0.079	0.116 (0.029; 0.202)	0.009 *	0.009 *	0.099	0.015 *	0.625
	16MB–26MB	0.001 (−0.032; 0.035)	0.940	0.140 (0.081; 0.199)	<0.001 *	0.177 (0.089; 0.264)	<0.001 *	0.117 (0.037; 0.198)	0.005 *	<.0.001 *	<0.001*	0.009 *	0.323
	16DB–26DB	−0.008 (−0.042; 0.026)	0.646	0.119 (0.058; 0.180)	<0.001 *	0.153 (0.063; 0.243)	0.001 *	0.096 (0.014; 0.178)	0.022 *	<0.001 *	0.001*	0.022 *	0.359
	33g–43g	−0.028 (−0.064; 0.008)	0.113	0.050 (0.011; 0.090)	0.014 *	0.032 (−0.025; 0.088)	0.272	0.080 (0.025; 0.136)	0.005 *	0.005 *	0.078	0.002 *	0.227
	33c–43c	−0.016 (−0.054; 0.022)	0.411	0.069 (0.027; 0.111)	0.002 *	0.046 (−0.015; 0.106)	0.136	0.100 (0.041; 0.159)	0.001 *	0.004 *	0.090	0.002 *	0.206
	36g–46g	−0.018 (−0.042; 0.007)	0.155	0.064 (0.021; 0.108)	0.004 *	0.055 (−0.010; 0.121)	0.094	0.071 (0.012; 0.131)	0.019 *	0.001 *	0.039*	0.007 *	0.724
	36f–46f	−0.012 (−0.038; 0.013)	0.344	0.113 (0.067; 0.158)	<0.001 *	0.135 (0.067; 0.204)	<0.001 *	0.090 (0.028; 0.152)	0.005 *	<0.001 *	<0.001*	0.003 *	0.333
	36MB–46MB	−0.018 (−0.047; 0.011)	0.218	0.110 (0.059; 0.160)	<0.001 *	0.137 (0.061; 0.212)	<0.001 *	0.083 (0.014; 0.152)	0.019 *	<0.001 *	<0.001*	0.009 *	0.300
	36DB–46DB	−0.020 (−0.052; 0.011)	0.212	0.123 (0.068; 0.178)	<0.001 *	0.142 (0.059; 0.224)	<0.001 *	0.105 (0.029; 0.180)	0.007 *	<0.001 *	<0.001*	0.003 *	0.517
	13g–23g	−0.019 (−0.145; 0.107)	0.765	−0.185 (−0.453; 0.083)	0.173	−0.152 (−0.588; 0.284)	0.490	−0.353 (−0.731; 0.024)	0.066	0.268	0.561	0.098	0.489
	13c–23c	0.036 (−0.068; 0.141)	0.494	−0.204 (−0.444; 0.036)	0.094	−0.374 (−0.736; −0.011)	0.043 *	−0.377 (−0.690; −0.063)	0.019 *	0.075	0.034 *	0.015 *	0.990
	16g–26g	0.038 (−0.047; 0.123)	0.379	0.047 (−0.121; 0.214)	0.582	−0.032 (−0.365; 0.300)	0.848	0.058 (−0.142; 0.259)	0.567	0.927	0.686	0.854	0.645
	16f–26f	0.025 (−0.077; 0.127)	0.627	−0.032 (−0.232; 0.168)	0.755	−0.070 (−0.467; 0.327)	0.728	−0.043 (−0.282; 0.197)	0.726	0.618	0.648	0.608	0.908
	16MB–26MB	0.053 (−0.047; 0.152)	0.296	−0.007 (−0.203; 0.189)	0.941	−0.096 (−0.484; 0.293)	0.628	0.003 (−0.232; 0.237)	0.981	0.589	0.466	0.699	0.669
	16DB–26DB	0.018 (−0.081; 0.118)	0.716	0.025 (−0.171; 0.222)	0.801	0.048 (−0.342; 0.439)	0.807	0.001 (−0.234; 0.236)	0.995	0.951	0.884	0.892	0.837
	33g–43g	−0.032 (−0.108; 0.044)	0.406	0.011 (−0.125; 0.147)	0.872	0.032 (−0.213; 0.277)	0.798	−0.054 (−0.233; 0.125)	0.553	0.586	0.624	0.824	0.578
	33c–43c	0.009 (−0.073; 0.090)	0.830	−0.072 (−0.218; 0.074)	0.329	−0.146 (−0.409; 0.116)	0.271	−0.082 (−0.274; 0.111)	0.401	0.338	0.265	0.391	0.694
	36g–46g	0.029 (−0.040; 0.098)	0.412	0.048 (−0.090; 0.186)	0.493	−0.199 (−0.468; 0.069)	0.144	0.150 (−0.012; 0.312)	0.069	0.807	0.106	0.175	0.029 *
	36f–46f	0.010 (−0.067; 0.087)	0.795	−0.041 (−0.193; 0.112)	0.599	−0.202 (−0.501; 0.097)	0.183	0.042 (−0.138; 0.223)	0.643	0.557	0.176	0.746	0.168
	36MB–46MB	0.029 (−0.056; 0.113)	0.504	−0.041 (−0.207; 0.126)	0.630	−0.128 (−0.456; 0.200)	0.442	0.019 (−0.178; 0.217)	0.848	0.465	0.363	0.932	0.449
	36DB–46DB	0.012 (−0.081; 0.104)	0.800	−0.039 (−0.221; 0.143)	0.670	−0.189 (−0.547; 0.170)	0.300	0.036 (−0.181; 0.252)	0.745	0.621	0.286	0.842	0.291
	13g–23g	−0.011 (−0.153; 0.130)	0.874	−0.188 (−0.493; 0.117)	0.224	0.019 (−0.447; 0.485)	0.935	−0.506 (−0.937; −0.074)	0.022 *	0.300	0.901	0.033 *	0.104
	13c–23c	0.033 (−0.086; 0.152)	0.587	−0.148 (−0.424; 0.127)	0.288	−0.008 (−0.400; 0.384)	0.967	−0.557 (−0.920; −0.193)	0.003 *	0.241	0.844	0.003 *	0.045
	16g–26g	0.044 (−0.052; 0.139)	0.366	0.008 (−0.169; 0.184)	0.933	0.164 (−0.144; 0.472)	0.294	−0.093 (−0.313; 0.128)	0.407	0.722	0.462	0.263	0.182
	16f–26f	0.048 (−0.066; 0.161)	0.407	−0.066 (−0.276; 0.144)	0.537	0.168 (−0.196; 0.533)	0.363	−0.212 (−0.473; 0.048)	0.110	0.350	0.532	0.073	0.096
	16MB–26MB	0.070 (−0.041; 0.181)	0.216	−0.021 (−0.227; 0.185)	0.841	0.206 (−0.152; 0.564)	0.257	−0.160 (−0.416; 0.096)	0.218	0.445	0.473	0.105	0.102
	16DB–26DB	0.050 (−0.060; 0.160)	0.370	−0.012 (−0.218; 0.194)	0.910	0.322 (−0.033; 0.678)	0.075	−0.199 (−0.452; 0.054)	0.121	0.603	0.150	0.076	0.019
	33g–43g	−0.048 (−0.133; 0.036)	0.259	−0.008 (−0.153; 0.136)	0.909	0.064 (−0.162; 0.289)	0.576	−0.111 (−0.307; 0.084)	0.261	0.637	0.358	0.559	0.247
	33c–43c	−0.008 (−0.099; 0.084)	0.868	−0.089 (−0.244; 0.066)	0.256	−0.045 (−0.289; 0.199)	0.714	−0.163 (−0.374; 0.049)	0.131	0.371	0.776	0.185	0.472
	36g–46g	0.051 (−0.029; 0.130)	0.210	0.022 (−0.125; 0.168)	0.770	−0.071 (−0.343; 0.200)	0.603	0.077 (−0.103; 0.258)	0.399	0.731	0.395	0.789	0.368
	36f–46f	0.046 (−0.042; 0.133)	0.302	−0.031 (−0.193; 0.131)	0.705	0.076 (−0.223; 0.375)	0.615	−0.046 (−0.245; 0.153)	0.649	0.410	0.848	0.406	0.503
	36MB–46MB	0.049 (−0.047; 0.144)	0.313	−0.039 (−0.215; 0.138)	0.665	0.163 (−0.163; 0.488)	0.324	−0.091 (−0.307; 0.126)	0.408	0.390	0.507	0.245	0.202
	36DB–46DB	0.063 (−0.042; 0.168)	0.240	−0.033 (−0.226; 0.159)	0.733	0.071 (−0.286; 0.429)	0.695	−0.052 (−0.290; 0.186)	0.666	0.388	0.965	0.385	0.572

**Table 6:** Relation between oral muscle pressure (tongue, lip, and cheek pressure and imbalance score) and the remaining occlusal characteristics. Within-group results for ordinal outcomes are presented as slopes indicating the change in dental outcome for a 1 unit increase in perioral muscle pressure. Positive slope values refer to positive relations (higher muscle pressure values for larger occlusal values). Within-group results for crossbite as the binary outcome are presented as odds ratios (OR) indicating the change in odds for crossbite for a 1 unit increase in perioral muscle pressure. OR > 1 refer to positive relations (higher muscle pressure values for higher odds of crossbite). Age and gender were included as covariates to account for possible confounding. *p*-values for differences between slopes or odds ratios between groups are presented in the last 4 columns. A probability level of <0.05 was considered significant (* *p* < 0.05).

		Control		CP + UCLP		CP		UCLP		Control vs. CP + UCLP	Control vs. CP	Control vs. UCLP	CP vs. UCLP
		Slope/OR (95% CI)	*p*-Value	Slope/OR (95% CI)	*p*-Value	Slope/OR (95% CI)	*p*-Value	Slope/OR (95% CI)	*p*-Value	Test for Difference of Slopes between Groups *p*-Value			
Effect of tongue pressure	Occlusion R	−0.002 (−0.008; 0.004)	0.471	−0.015 (−0.026; −0.005)	0.005 *	−0.018 (−0.038; 0.003)	0.086	−0.013 (−0.026; 0.000)	0.057	0.037 *	0.150	0.156	0.670
	Occlusion L	−0.003 (−0.010; 0.003)	0.303	−0.016 (−0.027; −0.005)	0.005 *	−0.016 (−0.037; 0.005)	0.135	−0.016 (−0.030; −0.002)	0.021 *	0.050 *	0.255	0.096	0.991
	Overjet	0.022 (−0.020; 0.065)	0.296	−0.029 (−0.103; 0.045)	0.439	−0.049 (−0.185; 0.087)	0.478	0.012 (−0.075; 0.098)	0.791	0.235	0.322	0.825	0.459
	Overbite	0.028 (−0.003; 0.060)	0.080	−0.004 (−0.060; 0.052)	0.886	−0.086 (−0.191; 0.019)	0.108	0.037 (−0.030; 0.104)	0.280	0.321	0.041 *	0.824	0.054
	Cross bite	1.000 (0.987; 1.030)	0.976	1.013 (0.956; −1.073)	0.663	1.011 (0.939; 1.088)	0.767	1.000 (0.972; 1.030)	0.976	0.708	0.933	0.916	0.791
Effect of lip pressure	Occlusion R	−0.007 (−0.022; 0.008)	0.355	0.031 (0.010; 0.053)	0.004 *	0.025 (−0.002; 0.052)	0.073	0.038 (0.003; 0.073)	0.032 *	0.004 *	0.044 *	0.020 *	0.547
	Occlusion L	−0.005 (−0.020; 0.011)	0.563	0.017 (−0.005; 0.040)	0.135	−0.002 (−0.030; 0.026)	0.895	0.048 (0.011; 0.084)	0.011 *	0.120	0.869	0.010 *	0.036 *
	Overjet	−0.108 (−0.209; −0.006)	0.037 *	0.069 (−0.076; 0.214)	0.350	0.061 (−0.118; 0.239)	0.504	0.020 (−0.211; 0.251)	0.864	0.050	0.105	0.316	0.785
	Overbite	0.006 (−0.072; 0.085)	0.874	−0.006 (−0.118; 0.106)	0.921	0.036 (−0.106; 0.179)	0.615	−0.091 (−0.274; 0.093)	0.332	0.863	0.716	0.339	0.283
	Cross bite	1.010 (0.943; 1.083)	0.773	1.066 (0.931; 1.221)	0.352	0.963 (0.806; 1.150)	0.675	1.010 (0.943; 1.083)	0.773	0.486	0.165	0.206	0.620
Effect of imbalance score	Occlusion R	−0.000 (−0.004; 0.004)	0.906	−0.012 (−0.018; −0.006)	<0.001 *	−0.010 (−0.018; −0.001)	0.025 *	−0.013 (−0.023; −0.004)	0.007 *	0.003 *	0.050	0.015 *	0.583
	Occlusion L	−0.002 (−0.007; 0.002)	0.320	−0.009 (-0.016; −0.003)	0.006 *	-0.004 (-0.013; 0.005)	0.411	−0.017 (−0.027; −0.007)	0.001 *	0.075	0.767	0.010 *	0.057
	Overjet	0.028 (−0.001; 0.057)	0.059	−0.022 (−0.065; 0.022)	0.324	−0.024 (−0.081; 0.033)	0.402	0.010 (−0.054; 0.075)	0.752	0.062	0.107	0.623	0.429
	Overbite	0.016 (−0.006; 0.038)	0.156	−0.008 (−0.041; 0.025)	0.635	−0.041 (−0.085; 0.003)	0.071	0.041 (−0.009; 0.091)	0.110	0.238	0.025 *	0.369	0.017 *
	Cross bite	0.998 (0.978; 1.018)	0.811	0.991 (0.958; 1.025)	0.880	1.020 (0.966; 1.077)	0.483	0.998 (0.978; 1.018)	0.811	0.730	0.155	0.252	0.459
Effect of right cheek pressure	Occlusion R	0.003 (−0.009; 0.015)	0.670	0.009 (−0.012; 0.031)	0.396	0.018 (−0.024; 0.061)	0.401	0.002 (−0.023; 0.028)	0.861	0.593	0.488	0.981	0.527
	Occlusion L	0.006 (−0.007; 0.018)	0.357	−0.002 (−0.024; 0.02.)	0.870	0.004 (−0.041; 0.048)	0.875	−0.005 (−0.032; 0.021)	0.695	0.554	0.923	0.456	0.737
	Overjet	0.025 (−0.056; 0.106)	0.543	−0.049 (−0.194; 0.096)	0.506	0.168 (−0.106; 0.442)	0.227	−0.181 (−0.343; −0.018)	0.029 *	0.380	0.322	0.025 *	0.032 *
	Overbite	0.018 (−0.043; 0.079)	0.560	0.011 (−0.100; 0.121)	0.851	0.167 (−0.050; 0.385)	0.130	−0.057 (−0.185; 0.072)	0.387	0.910	0.193	0.302	0.082
	Cross bite	0.991 (0.937; 1048)	0.754	1.145 (1.016; 1.289)	0.029 *	1.215 (1.020; 1.448)	0.030 *	0.991 (0.937; 1.048)	0.754	0.031 *	0.457	0.349	0.030 *
Effect of left cheek pressure	Occlusion R	0.008 (−0.005; 0.021)	0.240	0.015 (−0.007; 0.038)	0.187	0.017 (−0.023; 0.057)	0.393	0.010 (−0.018; 0.038)	0.486	0.590	0.665	0.905	0.766
	Occlusion L	0.010 (−0.004; 0.024)	0.147	0.009 (−0.014; 0.033)	0.435	0.023 (−0.019; 0.064)	0.276	0.001 (−0.028; 0.030)	0.934	0.994	0.568	0.582	0.399
	Overjet	0.073 (−0.017; 0.163)	0.112	−0.097 (−0.249; 0.056)	0.212	0.056 (−0.198; 0.310)	0.663	−0.238 (−0.417; −0.059)	0.010 *	0.060	0.902	0.002 *	0.064
	Overbite	0.065 (−0.004; 0.133)	0.063	0.018 (−0.097; 0.134)	0.754	0.203 (0.004; 0.403)	0.046 *	−0.087 (−0.228; 0.054)	0.224	0.500	0.196	0.057	0.020 *
	Cross bite	0.987 (0.927; 1.052)	0.694	1.095 (0.971; 1.234)	0.139	1.155 (0.983; 1.356)	0.079	0.987 (0.927; 1.052)	0.694	0.136	0.368	0.685	0.075

**Table 7:** Relation between oral muscle pressure (tongue, lip, and cheek pressure and imbalance score) and functional habits within and between the different groups, as well as the statistical significance. Results are presented as mean differences and 95% confidence intervals. Age and gender were included as covariates to account for possible confounding. A probability level of <0.05 was considered significant (* *p* < 0.05).

		Control		CP + UCLP		CP		UCLP		Control vs. CP + UCLP	Control vs. CP	Control vs. UCLP	CP vs. UCLP
		Mean Diff (95% CI)	*p*-Value	Mean Diff (95% CI)	*p*-Value	Mean Diff (95% CI)	*p*-Value	Mean Diff (95% CI)	*p*-Value	Test for Difference of Slopes between Groups *p*-Value			
Effect on tongue pressure	Nail biting	2.665 (−3.278; 8.607)	0.377	7.356 (−2.659; 17.372)	0.149	12.500 (−2.932; 27.932)	0.112	4.345 (−8.897; 17.588)	0.517	0.427	0.242	0.819	0.429
	Pen chewing	8.233 (−3.057; 19.52)	0.152	3.873 (−5.779; 13.524)	0.429	12.467 (−1.251; 26.184)	0.075	−2.065 (−16.46; 12.326)	0.777	0.562	0.637	0.266	0.151
	Thumb sucking	−0.822 (−20.11; 18.465)	0.933	−7.810 (−35.11; 19.488)	0.572	−5.200 (−33.08; 22.681)	0.713	-	-	0.680	0.799	-	-
	Bruxism	−1.430 (−10.02; 7.158)	0.742	13.300 (−2.699; 29.299)	0.103	-	-	12.083 (−4.317; 28.483)	0.147	0.221	-	0.151	-
	Infantile swallowing	−6.889 (−12.51; −1.266)	0.017 *	−4.685 (−13.64; 4.264)	0.302	−3.833 (−19.08; 11.411)	0.620	−5.520 (−16.65; 5.609)	0.328	0.681	0.711	0.829	0.860
	Lip interposition	−4.306 (−11.88; 3.266)	0.263	−6.951 (−26.38; 12.479)	0.480	−5.200 (−33.02; 22.625)	0.712	−7.538 (−34.99; 19.916)	0.588	0.802	0.951	0.823	0.906
	Physiological tongue position at rest	4.672 (−1.149; 10.494)	0.115	−3.818 (−11.81; 4.172)	0.346	−8.000 (−21.26; 5.256)	0.235	−1.429 (−11.45; 8.592)	0.778	0.092	0.086	0.299	0.435
	Caudal tongue position at rest	−0.36 (−6.501; 5.730)	0.901	3.780 (−4.403; 11.964)	0.362	8.000 (−5.400; 21.400)	0.240	1.633 (−8.746; 12.013)	0.756	0.421	0.262	0.741	0.459
	Interdental tongue position at rest	−8.157 (−16.13; 0.182)	0.045 *	−6.093 (−32.81; 20.621)	0.652	-	-	−8.000 (−34.84; 18.842)	0.556	0.884	-	0.991	-
Effect on lip pressure	Nail biting	2.456 (−0.233; 5.145)	0.073	−0.717 (−5.264; 3.830)	0.756	−1.417 (−8.440; 5.607)	0.691	−0.381 (−6.434; 5.672)	0.901	0.237	0.311	0.399	0.825
	Pen chewing	0.915 (−4.232; 6.062)	0.726	−2.344 (−6.760; 2.072)	0.296	−4.100 (−10.42; 2.216)	0.201	−1.545 (−8.194; 5.103)	0.646	0.344	0.226	0.564	0.582
	Thumb sucking	−5.600 (−14.20; 2.996)	0.200	−11.05 (−23.22; 1.120)	0.075	−12.33 (−24.75; 0.083)	0.052	-	-	0.471	0.379	-	-
	Bruxism	−1.782 (−5.690; 2.126)	0.369	−3.718 (−11.00; 3.569)	0.315	-	-	−3.362 (−10.85; 4.127)	0.376	0.644	-	0.712	-
	Infantile swallowing	−2.383 (−4.956; 0.190)	0.069	−2.150 (−6.264; 1.964)	0.303	−8.750 (−15.60; −1.902)	0.013 *	1.542 (−3.498; 6.581)	0.546	0.925	0.087	0.171	0.018 *
	Lip interposition	−2.563 (−5.970; 0.844)	0.139	−5.550 (−14.30; 3.197)	0.212	−12.33 (−24.77; 0.104)	0.052	0.720 (−11.56; 13.000)	0.908	0.530	0.136	0.611	0.142
	Physiological tongue position at rest	−0.500 (−3.199; 2.199)	0.714	1.58 (−2.689; 4.806)	0.577	3.875 (−2.274; 10.024)	0.215	−0.648 (−5.385; 4.089)	0.787	0.505	0.200	0.957	0.251
	Caudal tongue position at rest	1.108 (−1.704; 3.921)	0.437	−1.023 (−4.826; 2.780)	0.5954	−3.875 (−10.04; 2.287)	0.216	0.667 (−4.182; 5.515)	0.786	0.374	0.148	0.876	0.254
	Interdental tongue position at rest	−0.991 (−4.704; 2.721)	0.599	0.262 (−12.18; 12.700)	0.967	-	-	0.769 (−11.79; 13.326)	0.904	0.849	-	0.791	-
Effect on imbalance score	Nail biting	−2.435 (−11.59; 6.724)	0.600	8.624 (−6.863; 24.112)	0.273	15.676 (−8.115; 39.467)	0.195	4.490 (−16.02; 24.995)	0.666	0.226	0.162	0.543	0.482
	Pen chewing	10.036 (−7.217; 27.289)	0.252	11.237 (−3.566; 26.040)	0.136	24.810 (3.909; 45.711)	0.020 *	2.534 (−19.47; 24.534)	0.820	0.917	0.281	0.595	0.149
	Thumb sucking	18.940 (−10.25; 48.130)	0.202	30.505 (−10.82; 71.829)	0.147	36.585 (−5.414; 78.585)	0.087	-	-	0.652	0.496	-	-
	Bruxism	2.672 (−10.48; 15.818)	0.688	21.729 (−2.784; 46.242)	0.082	-	-	19.664 (−5.495; 44.823)	0.125	0.178	-	0.239	-
	Infantile swallowing	−3.152 (−11.98; 5.678)	0.481	1.507 (−12.61; 15.625)	0.833	22.810 (−0.661; 46.280)	0.057	−10.78 (−28.05; 6.497)	0.219	0.581	0.042 *	0.437	0.024 *
	Lip interposition	−0.455 (−12.11; 11.205)	0.939	12.339 (−17.60; 42.273)	0.416	36.585 (−5.818; 78.988)	0.090	−9.420 (−51.29; 32.449)	0.657	0.432	0.098	0.684	0.129
	Physiological tongue position at rest	7.432 (−1.604; 16.469)	0.106	−7.110 (−19.66; 5.440)	0.264	−20.81 (−41.19; −0.431)	0.045 *	1.234 (−14.47; 16.934)	0.877	0.065	0.013 *	0.498	0.092
	Caudal tongue position at rest	1.201 (−8.31; 10.711)	0.010 *	7.036 (−5.825; 19.897)	0.281	20.811 (0.166; 41.456)	0.048 *	−1.045 (−17.29; 15.199)	0.899	0.472	0.090	0.813	0.102
	Interdental tongue position at rest	−16.15 (−28.42; −3.866)	0.010 *	−6.862 (−48.00; 34.278)	0.742	-	-	−10.00 (−51.34; 31.329)	0.633	0.669	-	0.778	-
Effect on right cheek pressure	Nail biting	0.791 (−2.236; 3.819)	0.606	0.085 (−5.021; 5.191)	0.974	3.250 (−4.610; 11.110)	0.415	−2.582 (−9.327; 4.163)	0.450	0.814	0.565	0.368	0.267
	Pen chewing	2.674 (−3.081; 8.430)	0.360	−0.245 (−5.151; 4.661)	0.921	−2.100 (−9.138; 4.938)	0.556	0.348 (−7.036; 7.732)	0.926	0.446	0.301	0.624	0.636
	Thumb sucking	−2.044 (−11.60; 7.514)	0.673	−13.81 (−27.39; −0.230)	0.046 *	−15.80 (−29.61; −1.992)	0.025 *	-	-	0.164	0.108	-	-
	Bruxism	−2.840 (−7.190; 1.511)	0.199	−3.033 (−11.13; 5.061)	0.460	-	-	−2.292 (−10.58; 5.998)	0.585	0.967	-	0.908	-
	Infantile swallowing	1.004 (−1.875; 3.883)	0.492	−0.099 (−4.727; 4.528)	0.966	−6.083 (−13.86; 1.698)	0.124	3.263 (−2.417; 8.944)	0.258	0.690	0.093	0.484	0.057
	Lip interposition	1.633 (−2.147; 5.412)	0.394	−6.805 (−16.58; 2.972)	0.171	−15.80 (−29.63; −1.969)	0.026 *	1.346 (−12.30; 14.993)	0.846	0.114	0.018 *	0.968	0.083
	Physiological tongue position at rest	0.455 (−2.543; 3.452)	0.765	0.682 (−3.430; 4.794)	0.743	3.375 (−3.448; 10.198)	0.329	−0.857 (−6.015; 4.301)	0.743	0.930	0.439	0.664	0.329
	Caudal tongue position at rest	0.447 (−2.685; 3.579)	0.778	−0.726 (−4.913; 3.461)	0.732	−3.375 (−10.23; 3.478)	0.332	0.683 (−4.625; 5.992)	0.799	0.658	0.317	0.940	0.356
	Interdental tongue position at rest	−1.641 (−5.753; 2.472)	0.431	0.535 (−13.24; 14.307)	0.939	-	-	1.333 (−12.53; 15.200)	0.849	0.765	-	0.685	-
Effect on left cheek pressure	Nail biting	−0.448 (−3.118; 2.222)	0.740	−0.242 (−4.814; 4.330)	0.917	5.500 (−1.431; 12.431)	0.119	−4.809 (−10.76; 1.139)	0.112	0.939	0.116	0.188	0.027 *
	Pen chewing	1.709 (−3.410; 6.829)	0.510	0.388 (−4.009; 4.784)	0.862	−3.400 (−9.661; 2.861)	0.285	3.207 (−3.361; 9.774)	0.336	0.700	0.214	0.723	0.152
	Thumb sucking	−2.967 (−11.66; 5.723)	0.501	−2.357 (−14.67; 9.960)	0.706	−3.867 (−16.42; 8.687)	0.543	-	-	0.936	0.907	-	-
	Bruxism	−0.354 (−4.270; 3.562)	0.859	0.392 (−6.911; 7.694)	0.916	-	-	1.292 (−6.171; 8.755)	0.733	0.859	-	0.700	-
	Infantile swallowing	−0.067 (−2.653; 2.519)	0.959	−0.419 (−4.567; 3.728)	0.842	−5.167 (−12.16; 1.822)	0.146	2.283 (−2.819; 7.385)	0.378	0.887	0.178	0.418	0.091
	Lip interposition	1.033 (−2.412; 4.478)	0.554	−1.890 (−10.71; 6.927)	0.672	−3.867 (−16.47; 8.741)	0.545	−0.538 (−12.98; 11.902)	0.932	0.542	0.460	0.810	0.711
	Physiological tongue position at rest	0.687 (−1.982; 3.357)	0.611	−1.591 (−5.240; 2.058)	0.390	−1.500 (−7.576; 4.576)	0.626	−1.643 (−6.236; 2.950)	0.480	0.320	0.515	0.387	0.970
	Caudal tongue position at rest	1.158 (−1.621; 3.937)	0.411	1.585 (−2.119; 5.289)	0.399	1.500 (−4.581; 7.581)	0.626	1.467 (−3.244; 6.177)	0.539	0.855	0.920	0.911	0.993
	Interdental tongue position at rest	−3.322 (−6.943; 0.299)	0.072	−1.419 (−13.55; 10.709)	0.817	-	-	−0.704 (−12.91; 11.506)	0.909	0.767	-	0.685	-

## Data Availability

The data presented in this study are available on request from the corresponding author.
